# Quadrivalent Formulation of Intranasal Influenza Vaccine M2SR (M2-Deficient Single Replication) Protects against Drifted Influenza A and B Virus Challenge

**DOI:** 10.3390/vaccines11040798

**Published:** 2023-04-04

**Authors:** Lindsay Hill-Batorski, Yasuko Hatta, Michael J. Moser, Sally Sarawar, Gabriele Neumann, Yoshihiro Kawaoka, Pamuk Bilsel

**Affiliations:** 1 FluGen Inc., Madison, WI 53711, USA; 2 The Biomedical Research Institute of Southern California, Oceanside, CA 92056, USA; 3 Influenza Research Institute, Department of Pathobiological Sciences, School of Veterinary Medicine, University of Wisconsin, Madison, WI 53711, USA

**Keywords:** influenza, vaccine, quadrivalent, heterosubtypic, mouse, ferret

## Abstract

Current influenza vaccines demonstrate low vaccine efficacy, especially when the predominantly circulating strain and vaccine are mismatched. The novel influenza vaccine platform M2- or BM2-deficient single replication (M2SR and BM2SR) has been shown to safely induce strong systemic and mucosal antibody responses and provide protection against significantly drifted influenza strains. In this study, we demonstrate that both monovalent and quadrivalent (Quad) formulations of M2SR are non-pathogenic in mouse and ferret models, eliciting robust neutralizing and non-neutralizing serum antibody responses to all strains within the formulation. Following challenge with wildtype influenza strains, vaccinated mice and ferrets demonstrated reduced weight loss, decreased viral replication in the upper and lower airways, and enhanced survival as compared to mock control groups. Mice vaccinated with H1N1 M2SR were completely protected from heterosubtypic H3N2 challenge, and BM2SR vaccines provided sterilizing immunity to mice challenged with a cross-lineage influenza B virus. Heterosubtypic cross-protection was also seen in the ferret model, with M2SR vaccinated animals exhibiting decreased viral titers in nasal washes and lungs following the challenge. BM2SR-vaccinated ferrets elicited robust neutralizing antibodies toward significantly drifted past and future influenza B strains. Mice and ferrets that received quadrivalent M2SR were able to mount immune responses equivalent to those seen with each of the four monovalent vaccines, demonstrating the absence of strain interference in the commercially relevant quadrivalent formulation.

## 1. Introduction

Vaccination remains the best line of defense against seasonal influenza epidemics [1,2]. However, vaccine effectiveness (VE) for currently licensed vaccines is suboptimal and can vary widely, even in healthy, young adult populations [3]. One of the most well-documented factors to impact VE is antigenic drift, a continuous process of evolution that results in the rapid emergence of new, immunogenically unique virus strains. To combat antigenic drift, influenza vaccines are reformulated annually based on strain recommendations from the WHO, the CDC, and other collaborating surveillance centers [4]. However, the accurate prediction of emerging strains is complex and not infallible. When circulating viruses do not match the recommended vaccine strain, current vaccine candidates provide little to no protection. This occurred as recently as 2014–2015 when a poor match between the selected H3N2 vaccine strain and emergent viruses that predominantly circulated that season was associated with increased morbidity and mortality and a VE of as low as 6% [5,6,7]. Thus, there is a need for more broadly protective influenza vaccines able to induce robust, durable protection across multiple subtypes of influenza. Such vaccines may reduce or eliminate the need for yearly formulation updates and provide better protection against newly emerging potentially pandemic strains.

We have developed a novel vaccine platform that mimics wild-type influenza for a single-replication cycle when administered intranasally. This M2-deficient single-replication (M2SR) vaccine does not express the essential M2 ion channel protein and, thus, does not generate an infectious virus. An M2SR vaccine generated from A/Puerto Rico/8/34 (H1N1) protected mice against a lethal challenge from both homosubtypic and heterosubtypic (H3N2) influenza viruses [8]. Vaccination with an M2SR vaccine expressing A/California/07/2009 (H1N1pdm) HA and NA also provided heterosubtypic protection against the highly pathogenic H5N1 virus in mice and the gold standard ferret model [9]. Furthermore, we recently demonstrated that replication-deficient BM2 knockout (BM2SR) viruses expressing influenza B HA and NA proteins from both virus lineages circulating in humans (i.e., the Yamagata and Victoria lineages) were able to induce robust systemic and mucosal immune responses and provide cross-lineage protection from a lethal influenza B Victoria challenge in mice [10]. In contrast to current comparator influenza vaccines such as live-attenuated FluMist, the M2SR vaccine platform provides effective protection in ferrets with established influenza immunity [11]. In multiple clinical trials, the M2SR platform has been found to be safe and well-tolerated in humans. Moreover, the monovalent H3N2 M2SR induced robust influenza-specific serum and mucosal antibodies, and significant B and T cell responses, resulting in protection against drifted influenza A challenge [12,13,14].

Over the last decade, shifts to quadrivalent formulations have been made to improve the coverage and efficacy of the standard seasonal influenza vaccine. In the 2021–2022 season, despite different approvals for some age groups, all influenza vaccines available in the United States were quadrivalent. In the current study, we investigated whether a commercially relevant quadrivalent form of the M2SR vaccine (Quad M2SR) would elicit equivalent immunogenicity and provide protection against influenza challenge in both mouse and the gold standard ferret model. Additionally, utilizing sera from the ferret study, we explored the potential of our current Quad M2SR formulation to provide protective immune responses against both past and future drifted influenza strains.

## 2. Materials and Methods

### 2.1. Animals

Seven- to eight-week-old female BALB/c mice (Envigo, now Inotiv, Madison, WI, USA) and three- to five-month-old male ferrets (Marshall Farms, North Rose, NY, USA) were used. Ferret serum samples were tested to ensure that animals had not been exposed to influenza prior to initiating the study. All animal study protocols were approved by the FluGen, University of Wisconsin-Madison, or IIT Research Institute Institutional Animal Care and Use Committees, and all experiments were performed in accordance with the National Institute of Health guidelines for the care and use of laboratory animals.

### 2.2. Mouse Infection and Sample Collection

Groups of 22 mice were intranasally (IN) infected with a 10^6^ TCID_50_ dose of monovalent vaccines H1N1 M2SR (A/California/07/2009), H3N2 M2SR (A/Brisbane/10/2007), Yam BM2SR (B/Wisconsin/01/2010), or Vic BM2SR (B/Brisbane/60/2008), or a 10^6^ TCID_50_ dose of each of the four monovalent strains formulated together (Quad M2SR), or PBS (mock-infected). Twenty-eight days post prime vaccination, all mice received an equivalent boosting dose. Mice were weighed daily for 14 days and monitored for clinical symptoms following each vaccination. Serum was collected 3 weeks following boost vaccination. HA inhibition titers were determined by HAI assay as previously described [8]. Ten weeks post first vaccination, all mice were challenged with either a 40 MLD_50_ dose of A/Aichi/2/68 (H3N2) or a 20 MLD_50_ dose of B/Malaysia/2506/2004 (Victoria lineage), and body weight and survival were monitored for 14 days. A body-weight reduction of 25% or more that did not improve within 24 h was a criterion for humane euthanasia. On day 4 post-challenge, lungs and nasal turbinates were collected from 3 mice per group, and virus titers were determined by a plaque-forming assay in MDCK cells (Sigma-Aldrich, St. Louis, MO, USA, Cat#84121903), as previously described [15].

### 2.3. Ferret Infection and Sample Collection

Groups of 12 ferrets were intranasally inoculated with a 10^7^ TCID_50_ dose of H1N1 M2SR (A/California/07/2009), H3N2 M2SR (A/Brisbane/10/2007), Yam BM2SR (B/Wisconsin/01/2010), or Vic BM2SR (B/Brisbane/60/2008) or a 10^7^ TCID_50_ dose of each of the four monovalent strains (Quad M2SR), or PBS (mock-infected) (Table 1). Twenty-eight days post-vaccination, all ferrets received an equivalent boosting dose. Ferrets were weighed, and body temperature and clinical signs were observed for 7 days following each vaccination. Serum was collected pre-study, post-prime day 21, and post-boost day 35. HA-specific IgG and HA inhibition titers were determined by ELISA and HAI assay, respectively, as previously described [8]. Ten weeks post-first vaccination, all ferrets were challenged with a 10^6^ PFU dose of A/California/07/2009 (H1N1 pdm) influenza virus. Ferret body weight, body temperature, and clinical symptoms were monitored for 12 days post-challenge, and nasal washes were collected on days 1, 3, and 5 post-challenge. Three days post-challenge, 4 ferrets from groups that received PBS, Quad M2SR, and both H1N1 and H3N2 M2SRs were euthanized, and nasal turbinates, trachea, and lungs were collected for viral titer determination. Virus titers in nasal washes and organ homogenates were determined by a TCID_50_ assay in MDCK cells (Sigma-Aldrich, St. Louis, MO, USA, Cat#84121903), as previously described [16].

### 2.4. Virus Neutralization Assay

Pre-study and treatment phase serum samples (from days 21 and 35) were tested against 3 influenza B Victoria lineage viruses B/Malaysia/2506/2004, B/Brisbane/60/2008, and B/Wisconsin/30/2019 and 3 Influenza B Yamagata lineage viruses B/Florida/4/2006, B/Wisconsin/01/2010, and B/Wisconsin/28/2019 in a virus neutralization assay. The serum samples were inactivated at 56 °C for 1 h. The sera were serially diluted 2-fold and incubated with a standardized virus (concentration of 400–600 PFU) at 37 ± 2 °C in 5.0 ± 1% CO_2_ for 60 min. One hundred microliters (100 μL) of each serum and virus mixture was transferred into the respective wells of a 96-well plate containing a monolayer of MDCK cells (International Reagent Resource, Atlanta, GA, USA, Cat#FR-926) and layered with 1% methylcellulose. Cells were incubated for 18–22 h at 37 ± 2 °C in 5.0 ± 1% CO_2_. After incubation, the cells were fixed with paraformaldehyde and stained with an anti-influenza B nucleoprotein monoclonal antibody pool (Millipore; Billerica, MA, USA, or Invitrogen; Waltham, MA, USA), followed by peroxidase-conjugated goat anti-mouse IgG. The spots were developed using TrueBlue Peroxidase Substrate (KPL; Gaithersburg, MD, USA). The plaques were visualized and counted using an Immunospot instrument (CTL; Shaker Heights, OH, USA). The 50% plaque reduction neutralization titer (PRNT_50_) was calculated by counting plaques and reporting the titer as the reciprocal of the last serum dilution to show a 50% reduction in the input control virus plaque count based on the back-titration of control plaque.

## 3. Results

### 3.1. Quadrivalent M2SR Is Safe In Vivo and Elicits Functional Antibody Responses to Each Component within the Multivalent Formulation

**Figure 1:**
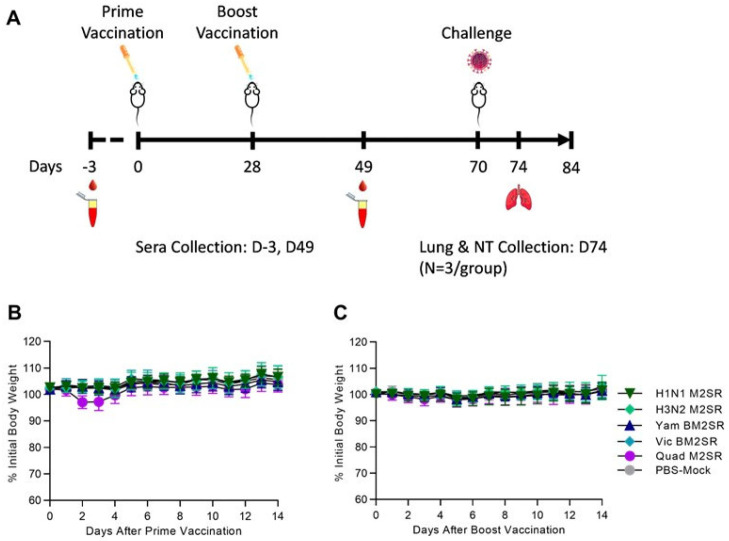
Vaccination scheme, sampling regimen, and body weight changes in mice following M2SR vaccination. (**A**) Groups of BALB/c were intranasally vaccinated with H1N1 M2SR (A/California/07/2009), H3N2 M2SR (A/Brisbane/10/2007), Yam BM2SR (B/Wisconsin/01/2010), Vic BM2SR (B/Brisbane/60/2008), or each of the four monovalent strains formulated together (Quad M2SR) on Day 0 and again on Day 28. Serum samples were collected pre-study and on day 49. Mice were challenged with a 40 MLD_50_ dose of A/Aichi/2/68 (H3N2) or a 20 MLD_50_ dose of B/Malaysia/2506/2004 (Victoria lineage) on Day 70. Lung and nasal turbinates were collected from 3 mice/group on Day 74. Body weight was monitored for 14 days following prime inoculation (**B**), and again 28 days later following boost inoculation (**C**).

Mice were intranasally inoculated on study day 0 (prime) and 28 (boost) with 10^6^ TCID_50_ of H1N1 M2SR, H3N2 M2SR, Vic BM2SR, Yam BM2SR, a combination of all four monovalent strains (Quad M2SR), or PBS control (mock). All mice received an equivalent boosting dose 4 weeks post prime vaccination (Figure 1A). Little weight loss (less than 3%) was seen in any group following prime or boost vaccinations (Figure 1B and Figure 1C, respectively), suggesting that both monovalent and quadrivalent forms of the M2SR vaccine are well tolerated and non-pathogenic in vivo. No mice exhibited any signs of illness.

**Figure 2:**
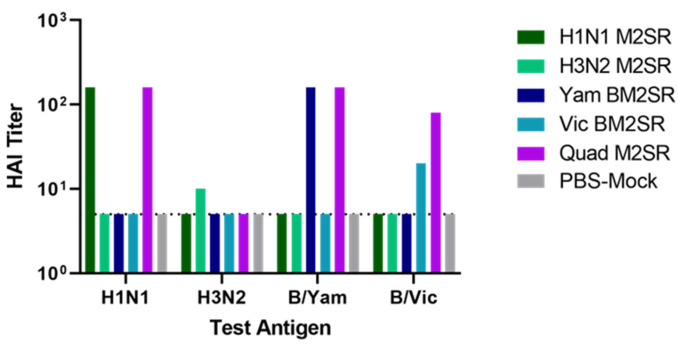
Serum antibody responses in mice following M2SR vaccination. Serum samples were collected from immunized mice 3 weeks following boost inoculation. HAI titers against analogous H1N1, H3N2, B/Yam, and B/Vic viral antigens were determined for pooled, RDE-treated sera. The HAI detection limit was five and is indicated by a dashed line.

Serum samples were collected post-boost inoculation, and functional immunogenicity was evaluated by a hemagglutination inhibition (HAI) assay. Pre-study serum HAI values against H1N1, H3N2, Influenza B-Vic, and Influenza B-Yam were confirmed to be below baseline at the study start (data not shown). As shown in Figure 2, strain-specific HAI levels were significantly increased after two doses of each monovalent M2SR. Quad M2SR elicited HAI titers that were within two-fold of levels seen after monovalent vaccination, except in the case of Vic BM2SR, where HAI values for Quad M2SR were 4-fold higher than its monovalent counterpart. Immunogenicity toward A/Brisbane/10/2007 H3N2 antigen was low or undetectable for both H3N2 monovalent and Quad M2SR. The PBS mock control group did not elicit any detectable HAI antibodies against any of the four strains tested. These data indicate that Quad M2SR induces functional HAI antibodies against multiple strains of influenza virus at levels similar to or better than monovalent vaccines.

### 3.2. Quadrivalent M2SR Protects Mice against Lethal Influenza A and B Challenge

**Figure 3:**
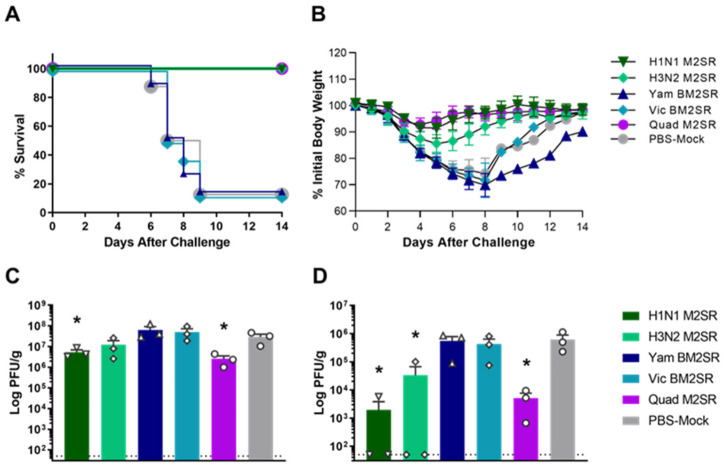
Survival and organ titers of M2SR vaccinated mice following challenge with H3N2 influenza virus. Mice were vaccinated with the indicated vaccines, as described in Materials and Methods. Six weeks following the final vaccination, mice were challenged with 40 MLD_50_ of live, wildtype A/Aichi/2/68 (H3N2). Survival (**A**) and body weight changes (**B**) were monitored for 14 days after the challenge. Lung (**C**) and nasal turbinates (**D**) were collected from 3 mice per group on day 4 post-challenge. Viral loads in samples were determined per gram by a plaque-forming assay, and the open faced symbols (e.g., triangles, diamonds, circles, etc.) represent virus titers from individual mice within the group, while colored bars represent the mean titer for the indicated group. The detection limit of the assay was 50 PFU/g and is shown with a horizontal dashed line. Asterisks indicate groups that were significantly different from PBS-mock groups: * *p* < 0.05 as compared by unpaired *t* test.

Mice prime-boost vaccinated with H1N1 M2SR, H3N2 M2SR, Vic BM2SR, Yam BM2SR, Quad M2SR, or PBS-mock were challenged with 40 MLD_50_ of A/Aichi/2/1968 (H3N2), which is antigenically distinct from the H3N2 M2SR (A/Brisbane/10/2007). As seen in Figure 3A, only 12.5% (1/8) of PBS-mock immunized mice survived the lethal A/Aichi/2/1968 challenge. Similarly, only 12.5% (1/8) of the mice vaccinated with either BM2SR monovalent vaccine survived. Mice vaccinated with H3N2 M2SR dipped in weight between 4 and 5 days post challenge (Figure 3B), however all H3N2 M2SR vaccinated mice (8/8) survived the challenge with the H3N2 drifted strain (Figure 3A). Additionally, 100% of mice (8/8) vaccinated with Quad M2SR survived the challenge with nominal weight loss, indicating that the quadrivalent vaccine is as effective at protecting mice from lethality as monovalent forms. All the mice (8/8) vaccinated with H1N1 M2SR also survived the challenge with the H3N2 virus suggesting the M2SR vaccine can protect mice across different subtypes of influenza A virus (Figure 3A). These findings were further reflected in viral titers recovered from the lungs and nasal turbinates of mice post-challenge with A/Aichi/2/1968 (Figure 3C and Figure 3D, respectively). Mice that received H1N1 M2SR, H3N2 M2SR, or Quad M2SR each had decreased lung and nasal turbinate viral titers post-challenge with the H3N2 virus as compared to mice who received monovalent BM2SRs or PBS.

**Figure 4:**
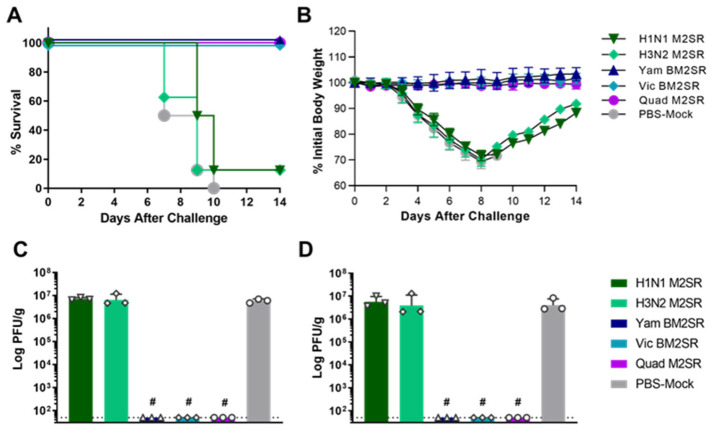
Survival and organ titers of M2SR vaccinated mice following challenge with B/Victoria influenza virus. Mice were vaccinated with indicated vaccines, as described in Materials and Methods. Six weeks following the final vaccination, mice were challenged with 20 MLD_50_ of live, wildtype B/Malaysia/2506/2004 (Victoria lineage). Survival (**A**) and body weight changes (**B**) were monitored for 14 days after the challenge. Lung (**C**) and nasal turbinates (**D**) were collected from 3 mice per group on day 4 post-challenge. Viral loads in samples were determined by a plaque-forming assay, and the open faced symbols (e.g., triangles, diamonds, circles, etc.) represent virus titers from individual mice within the group, while colored bars represent the mean titer for the indicated group. The detection limit of the assay was 50 PFU/g and is shown with a horizontal dashed line. Groups that were significantly different from PBS-mock groups are indicated: # *p* < 0.0005 as compared by unpaired *t* test.

The protective efficacy of Quad M2SR against influenza B was evaluated by challenging each group with 20 MLD_50_ of B/Malaysia/2506/2004 (Victoria lineage). As shown in Figure 4A, no PBS-mock immunized mice survived the lethal B/Malaysia/2506/2004 challenge. Similarly, only 12.5% (1/8) of mice vaccinated with either H1N1 or H3N2 monovalent vaccines survived the influenza B challenge. However, all mice vaccinated with Vic BM2SR survived the challenge with the antigenically drifted strain. Quadrivalent M2SR was also 100% protective following the B/Malaysia challenge, providing further evidence that Quad M2SR is efficacious at protecting against both influenza A and B viruses. Vaccination with Yam BM2SR also protected 100% of mice (8/8) against a challenge with the Victoria lineage virus, indicating cross-lineage protection following BM2SR vaccination (Figure 4A). Mice who received Vic, Yam or Quad BM2SR displayed no weight loss following B/Malaysia Challenge (Figure 4B). In addition, both monovalent BM2SRs, as well as the Quad M2SR, provided sterile immunity against the influenza B challenge, as demonstrated by the lack of detectable virus recovered from lungs or nasal turbinates on day 4 post-challenge (Figure 4C and Figure 4D, respectively). In contrast, mice who only received influenza A M2SR or PBS showed high titers of the challenge virus in the lungs and nasal turbinates.

### 3.3. Quadrivalent M2SR Is Well Tolerated in the Ferret Model

**Figure 5:**
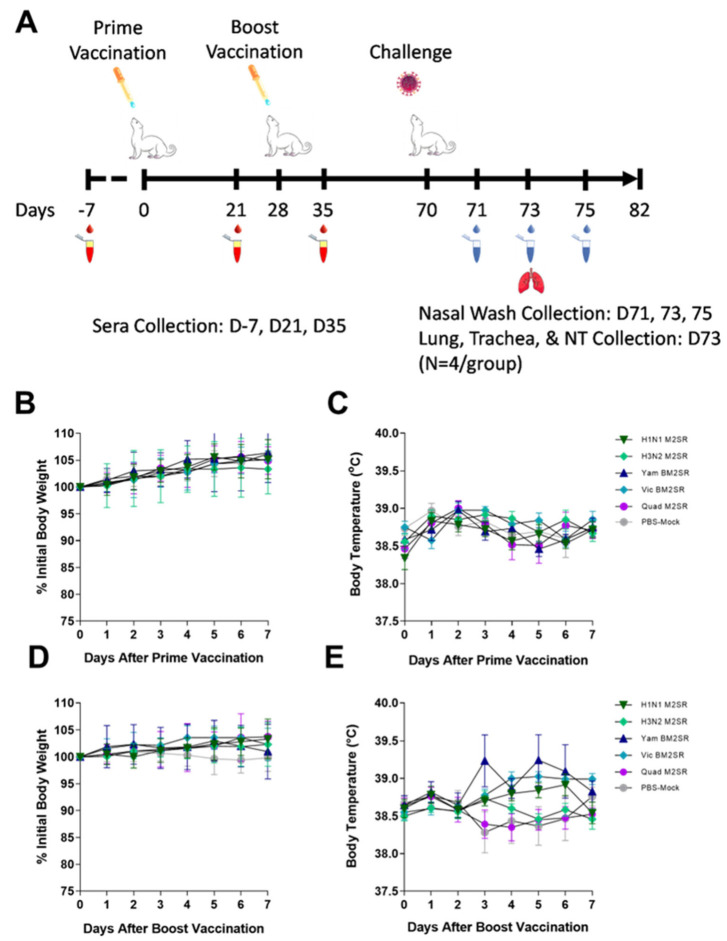
Vaccination scheme, sampling regimen, body weight, and temperature changes in ferrets following M2SR vaccination. (**A**) Groups of ferrets were intranasally vaccinated with H1N1 M2SR (A/California/07/2009), H3N2 M2SR (A/Brisbane/10/2007), Yam BM2SR (B/Wisconsin/01/2010), Vic BM2SR (B/Brisbane/60/2008), or each of the four monovalent strains formulated together (Quad M2SR) on Day 0 and again on Day 28. Serum samples were collected pre-study and on days 21 and 35. Ferrets were challenged with a 10^6^ PFU dose of A/California/07/2009 (H1N1 pdm) on Day 70. Nasal washes were taken on days 71, 73, and 75, and lung trachea and nasal turbinates were collected from 4 ferrets/group on Day 73. Body weight and temperature were monitored for 7 days following prime inoculation ((**B**,**C**), respectively) and again 28 days later following boost inoculation ((**D**,**E**), respectively).

Three- to five-month-old male naïve ferrets were intranasally inoculated with a 10^7^ TCID_50_ dose of H1N1 M2SR, H3N2 M2SR, Vic BM2SR, Yam BM2SR, a combination of all four monovalent strains (Quad M2SR), or PBS (mock-infected). Twenty-eight days post-vaccination, ferrets received an equivalent boosting dose (Figure 5A). All vaccine candidates, including Quad M2SR, were safe and well-tolerated, as no significant body weight or temperature changes were detected in any M2SR group or the PBS group (mock-infected) after one or two doses (Figure 5B,C and Figure 5D,E respectively).

### 3.4. Quadrivalent M2SR Elicits Antibody Responses toward all 4 Influenza Strains in the Ferret Model

**Figure 6:**
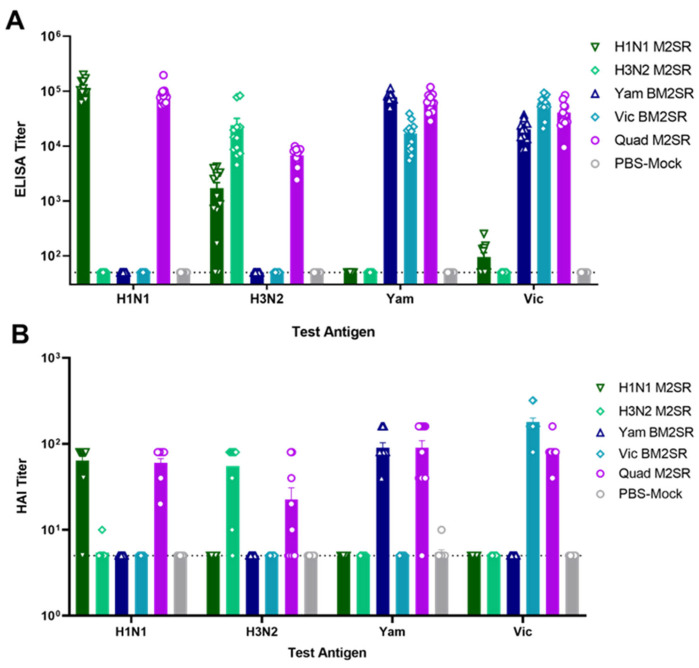
Serum antibody responses in ferrets following M2SR vaccination. Serum samples were collected from immunized ferrets 1 week following boost inoculation. ELISA titers (**A**) against analogous H1N1, H3N2, B/Yam, and B/Vic HA antigens were determined. HAI titers (**B**) against these viruses were determined for RDE-treated sera. Open faced symbols (e.g., triangles, diamonds, circles, etc.) represent titers from individual mice within the group, while colored bars represent the mean titer for the indicated group.The ELISA and HAI detection limits were 50 and 5, respectively, and indicated by a dashed line.

Ferret sera were collected and analyzed for IgG antibody titers against the HA of A/California/07/09 (H1N1), A/Brisbane/10/2007(H3N2), B/Wisconsin/01/010 (B/Yam), and B/Brisbane/60/2008 (B/Vic) by ELISA. Titers for all four strains before immunization were near baseline (data not shown). Robust serum IgG responses were seen in each monovalent group against the vaccine-matched antigens, while responses from PBS-treated animals remained at baseline (Figure 6A). Cross-lineage responses were seen following vaccination with either Vic or Yam BM2SR. The magnitude of antibody production from Quad M2SR was comparable to each monovalent, indicating that the quadrivalent formulation does not display strain interference in ferrets.

Functional immunogenicity was also determined via HAI assay against wildtype influenza viruses matched to the vaccine strains. Similar to the ELISA results, all vaccines were able to elevate HAI titers significantly from baseline (Figure 6B). PBS control groups did not elicit any HAI antibodies (Figure 6B). As expected, HAI antibody responses were more lineage-specific than total IgG following BM2SR vaccination. Importantly, all increases were comparable across all vaccine formulations, again indicating that the Quad M2SR formulation had no significant effect on individual monovalent performance. 

### 3.5. Quadrivalent M2SR Protects Ferrets against H1N1pdm Influenza a Virus Challenge

**Figure 7:**
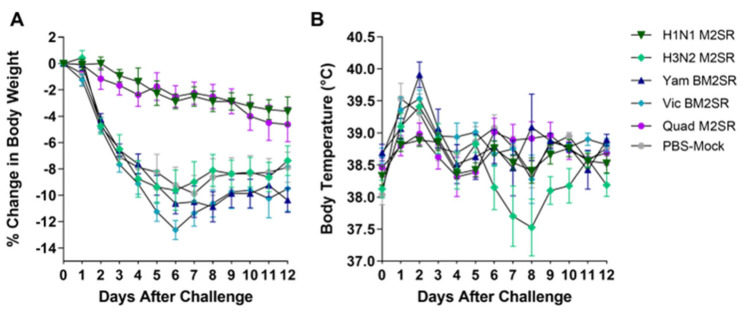
Body weight and temperature changes in M2SR vaccinated ferrets following a challenge with the H1N1 influenza virus. Ferrets were vaccinated with indicated vaccines, as described in Materials and Methods. Six weeks following the final vaccination, ferrets were challenged with the live, wildtype A/California/07/2009 (H1N1 pdm) influenza virus. Body weight (**A**) and temperature (**B**) were monitored for 12 days following the challenge.

Six weeks following boost vaccination, ferrets were challenged with a 10^6^ dose of H1N1 pandemic virus A/California/07/2009, and body weights and temperatures were monitored for 12 days. Both H1N1 M2SR and Quad M2SR provided protection against the challenge, as shown by decreased fever and minimal body weight loss (maximum 3.6–4.6%), as compared to the mock-infected group (maximum 9.6%, Figure 7).

**Figure 8:**
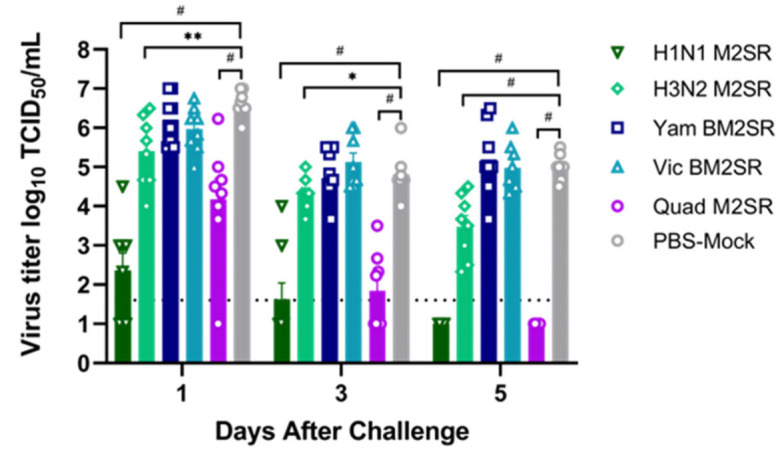
Nasal wash titers from M2SR-vaccinated ferrets following the challenge with the H1N1 influenza virus. Nasal washes were collected on days 1, 3, and 5 post-challenge. Virus loads in samples were determined by a TCID_50_ assay in MDCK cells. Open faced symbols (e.g., triangles, diamonds, circles, etc.) represent virus titers from individual mice within the group, while colored bars represent the mean titer for the indicated group. The detection limit of the assay was 1.5 log_10_ TCID_50_/mL, as indicated by a horizontal dotted line. Solid symbols above horizontal black bars (e.g., *, #) indicate results that were significantly different from those of the PBS-Mock group: * *p* < 0.05, ** *p* < 0.005, and # *p* < 0.0005, as compared by an unpaired *t* test.

On days 1, 3, and 5 post-challenge, nasal washes were performed on each group, and viral titer was assessed. When compared to the PBS control group, a significant reduction in nasal wash titers of 4.1 and 2.4 log10 TCID_50_/mL was observed on day 1 post-challenge in ferrets given H1N1 M2SR (*p* < 0.0001) and Quad M2SR (*p* = 0.0002), respectively (Figure 8). A significant reduction in titer was also seen in the H1N1 M2SR (*p* < 0.0001) and Quad M2SR (*p* < 0.0001) groups on day 3 post-challenge, with fewer animals exhibiting upper respiratory tract viral shedding (2/8 for H1N1 M2SR and 4/8 for Quad M2SR) compared to the PBS control group (8/8). By day 5 post-challenge, titers remained high in the control group, while no virus was detected in the nasal washes of ferrets who received H1N1 M2SR (*p* < 0.0001) or Quad M2SR (*p* < 0.0001). Interestingly, ferrets who received the H3N2 M2SR vaccine also exhibited significant reductions in viral titers on days 1, 3, and 5 post-challenge (*p* = 0.0013, 0.0273, and 0.0001, respectively), further suggesting heterosubtypic cross-protection by M2SR (Figure 8).

**Figure 9:**
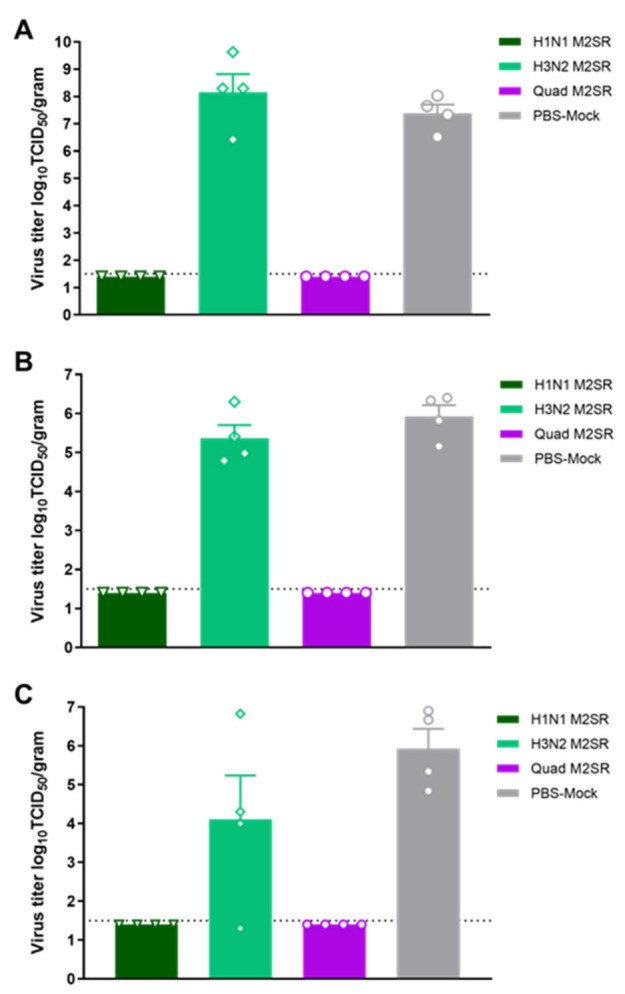
Organ titers from M2SR vaccinated ferrets following a challenge with the H1N1 influenza virus. Nasal turbinates (**A**), trachea (**B**), and lung (**C**) were collected from 4 ferrets per group on day 3 post-challenge. Virus loads in samples were determined by a TCID_50_ assay in MDCK cells. Open faced symbols (e.g., triangles, diamonds, circles, etc.) represent virus titers from individual mice within the group, while colored bars represent the mean titer for the indicated group. The detection limit of the assay was 1.5 log_10_ TCID_50_/mL, as indicated by a horizontal dotted line.

Further evidence of the protection provided by Quad M2SR vaccination was seen following an assessment of organ viral titer on day 3 post-challenge. Although viral titers were high in tissue collected from ferrets administered PBS, the virus was not detected in the nasal turbinates, trachea, or lungs of any animal who received the H1N1 M2SR or Quad M2SR vaccination (Figure 9). Similar to the nasal wash results, animals who received the H3N2 M2SR vaccine trended toward suppressed virus spread in the lower respiratory tract, exhibiting a 1.8 log decrease in mean titer as compared to the PBS group (*p* = 0.09), with 1 of 4 animals below the level of detection (Figure 9C). 

### 3.6. Quadrivalent M2SR Elicits Robust Neutralizing Antibody Responses against Past- and Future-Drifted Influenza B Strains

**Figure 10:**
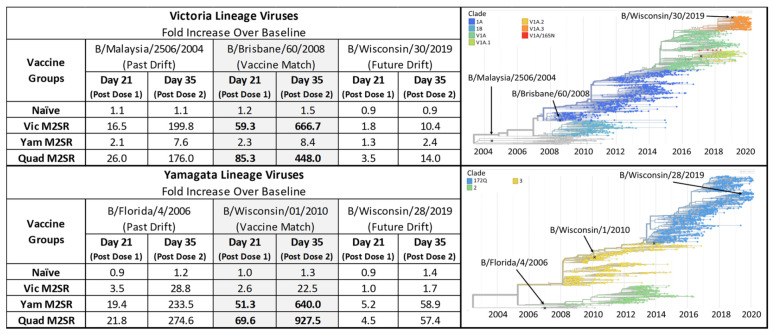
Fold increase serum antibody responses from BM2SR vaccinated ferrets against drifted influenza B strains. Serum was collected pre-study and after 1 and 2 doses of the indicated BM2SR vaccination. Samples were tested against 3 influenza B Victoria lineage viruses (B/Malaysia/2506/2004, B/Brisbane/60/2008, and B/Wisconsin/30/2019) and 3 influenza B Yamagata lineage viruses (B/Florida/4/2006, B/Wisconsin/01/2010, and B/Wisconsin/28/2019). PRNT_50_ titers were determined at each time point and averaged for the group. Values above represent the fold change in PRNT_50_ titer over pre-study values (baseline). Influenza B Victoria and Yamagata-lineage hemagglutinin phylogenetic tree and clades are shown with the location of the tested strains indicated by arrows. Color coding and clade designations are taken from Nextstrain.org [17].

Neutralizing antibody titers against influenza B viruses for each vaccine group were determined by a plaque reduction neutralization test (PRNT_50_). Ferret sera were subjected to PRNT_50_ analysis with vaccine-matched and drifted Yamagata or Victoria lineage influenza B viruses. As shown in Figure 10, robust neutralizing antibodies were produced against the vaccine-matched virus for both monovalent Vic BM2SR and Yam BM2SR after one dose, that was boosted more than 10-fold after the second dose. Both vaccines also cross-reacted well against past- and future-drifted viruses within their matching lineages. Quadrivalent M2SR elicited robust neutralizing antibodies toward past- and future-drifted strains of both influenza B lineages and demonstrated substantial increases following a boosting dose (Figure 10). Additionally, immunogenicity toward both lineages was equivalent to the monovalent formulations, further demonstrating the absence of strain interference in the quadrivalent formulation.

## 4. Discussion

Herein, we present evidence that the novel intranasal influenza vaccine, M2SR, provides safe and effective protection against homologous and heterologous influenza viruses in both monovalent and quadrivalent formulations. The Quad M2SR vaccine protected against influenza A H3N2 and influenza B challenge viruses that did not match the strains in the vaccine, demonstrating that M2SR maintains its broadly-reactive characteristics as a multivalent formulation.

Consistent with previous data, M2SR vaccines were well tolerated, with no weight loss or clinical signs of illness observed in mice who received one or two doses of monovalent or quadrivalent formulations, as compared to mock-infected groups. Similar safety profiles were evident in the ferret model, where temperature and body weights remained similar to control groups following single and prime-boost monovalent and quadrivalent immunization schedules.

Until 2012, influenza vaccines have been predominantly trivalent, containing representative H1N1 and H3N2 strains together with a single influenza B virus of either Victoria or Yamagata lineage, predicted to be dominant in that year. Due to little cross-reactive protection between the lineages, vaccine mismatch was responsible for little to no protection from influenza B epidemics by trivalent vaccines in 5 of the 10 influenza seasons from 2001 through 2010 [18]. Here, we showed that the Quad M2SR, which included two monovalent BM2SR vaccines representing both lineages, provided strong, sterilizing immunity against challenges with a mismatched influenza B virus. Specifically, inoculation of mice with the Quad M2SR vaccine provided complete protection from a drifted Victoria challenge, while eliminating viral spread in the upper and lower respiratory tract, demonstrating that Quad M2SR is capable of protection against both lineages. The cross-lineage protection observed following Yam BM2SR vaccination and B/Malaysia (Vic) challenge was not correlated to cross-strain HAI production, suggesting the mode of protection by BM2SR is not entirely due to HA neutralization. Previous literature has highlighted cross-lineage protection in the absence of cross-lineage serum antibody responses and provided evidence that local and/or cellular immune responses may play a role in this type of protection [19,20]. Indeed, we have previously shown intranasal M2SR to stimulate robust mucosal and strong, virus-specific T-cell responses that likely contribute to this cross-lineage protection [8,9,13,14].

Interestingly, broad cross-protection was not limited to BM2SR vaccines but was also observed in mouse and ferret models following vaccination with H1N1 and H3N2 monovalent M2SR formulations. Following the challenge with a highly drifted H3N2 strain, 100% survival and decreased viral titers in lungs and nasal turbinates were seen in mice who received either H1N1 or H3N2 M2SR. This is complementary to previously published mouse studies where H1N1 M2SR vaccination provided complete protection from a H3N2 and H5N1 challenge, respectively [8,9]. Heterosubtypic protection was also observed in the ferret model following a challenge with an H1N1 strain. Ferrets who received only H3N2 M2SR had decreased nasal wash titers on days 1, 3, and 5 and a trend toward decreased lung titers following a challenge with an H1N1 virus, as compared to the mock-infected group. Again, even though M2SR elicited robust homologous HAI antibodies, cross-subtype HA neutralizing antibodies were below levels typically correlated to protection, indicating a different mechanism for M2SR heterosubtypic cross-protection. Live-attenuated influenza vaccine trials have indicated that the HAI titer underestimates protection, which better correlates with local IgA production and T-cell responses [21,22,23]. Interestingly, we have recently shown that for M2SRs, consistent heterosubtypic cross-protection was dependent upon B cells and that T cell depletion had no effect on the survival of challenged mice [24]. 

Despite the demonstrated effectiveness of monovalent M2SR formulations, quadrivalent influenza vaccine formulations containing representatives from H1N1, H3N2, B/Vic, and B/Yam strains are likely the best-suited candidates for reducing influenza morbidity [25]. However, the inclusion of additional antigens or vaccine viruses in multivalent conformations raises considerations of interference and its potential to reduce immune responses to individual antigens. Viral interference among wild-type influenza viruses is well-documented, and interference among strains contained in the 2014/15 influenza vaccine FluMist was a potential factor underlying its decreased effectiveness and the recommendation against its use in two subsequent seasons [21]. In contrast, we showed here that Quad M2SR is as effective at generating antigen-specific immune responses as each of the four individual monovalent strains. Mice that received Quad M2SR generated functional HAI antibody levels equivalent to or within 2-fold of mice that received individual strains, except in the case of B/Brisbane/60/2008 antigen, where Quad M2SR elicited HAI levels 4-fold higher than its monovalent counterpart. Quad M2SR and H3N2 M2SR immunogenicity toward A/Brisbane/10/2007 H3N2 antigen was low. Yet both formulations were 100% effective in protecting mice from a H3N2 challenge, suggesting mechanisms behind M2SR protection are complex and not exclusively based on serum antibodies to the head region of HA. Signs of strain interference were also absent in ferrets that received the Quad M2SR formulation. Total IgG titers in the serum of ferrets who received Quad M2SR were robust and equivalent to those elicited by each monovalent group. Functional HAI titers following Quad M2SR vaccination were also within 2.4-fold of levels elicited by monovalent formulations, demonstrating Quad M2SR as being robustly immunogenic and devoid of strain interference. 

To account for continuous antigenic drift, compositions of influenza vaccines are revised yearly based on circulating viruses that are identified via continuous monitoring by the World Health Organization’s Global Influenza Surveillance and Response System. However, multiple challenges, such as the up to 6-month long duration needed for vaccine development, sometimes prevent accurate strain selection, resulting in mismatches between the recommended vaccine and circulating influenza strains. Here, we showed that monovalent and quadrivalent M2SR formulations can elicit broad immune responses against antigenically diverse viral strains in the ferret model. Following a single dose, both Yam and Vic BM2SR induced robust neutralizing antibody responses against their respective vaccine-matched strains. Similarly, high responses were seen from both monovalent formulations against strains within their lineage that circulated 4 years prior. Remarkably, a single dose of Yam BM2SR was able to generate a significant neutralizing response to a future Yamagata strain B/Wisconsin/28/2019 that appeared nearly 10 years later. Vic BM2SR performed less well against the more modern Victorian strain B/Wisconsin/30/2019. However, that response was significantly higher in ferrets who received the prime-boost vaccine regime. As seen previously, neutralizing antibodies elicited from a single or prime-boost dose of Quad M2SR were as high or higher than monovalent formulations for both lineages. These data suggest that M2SR is able to provide a broad, neutralizing immune response against antigenically diverse strains without the need for continual strain revision. 

## 5. Conclusions

In summary, our data demonstrated that monovalent and quadrivalent formulations of M2SR vaccination provide safe and effective protection against vaccine-matched and drifted influenza virus challenges in mice and ferrets.

## Figures and Tables

**Table 1:** M2SR Vaccine Candidates for Ferret Study.

Vaccine	HA and NA Components	Dose
H1N1 M2SR	A/California/07/2009	1 × 10^7^ TCID_50_
H3N2 M2SR	A/Brisbane/10/2007	1 × 10^7^ TCID_50_
Vic M2SR	B/Brisbane/60/2008	1 × 10^7^ TCID_50_
Yam M2SR	B/Wisconsin/01/2010	1 × 10^7^ TCID_50_
M2SR Quad	A/California/07/2009	4 × 10^7^ TCID_50_
	A/Brisbane/10/2007	
	B/Brisbane/60/2008	
	B/Wisconsin/01/2010	

## Data Availability

The data presented in this study are available on request from the corresponding author.
